# CT-guided intratumoral immunotherapy for advanced solid tumors: a prospective clinical study of safety and systemic antitumor effects

**DOI:** 10.3389/fimmu.2026.1869154

**Published:** 2026-07-24

**Authors:** Yongqiong Ou, Jian Zhang, Hongye Tan, Binjia He, Tianheng Li, Manting Liu, Cheng Zhi, Junhao Huang, Ming Li, Shenghua Zuo, Noor Ul Huda Shah, Yuning Chen, Junjian Huang, Dongni Chen, Ruzhai Qin, Xufeng Li, Hui Lian, Qingde Wu, Hainan Yang, Zhenfeng Zhang

**Affiliations:** 1 Department of Radiology; Translational Medicine Center; Guangzhou Key Laboratory for Research and Development of Nano-Biomedical Technology for Diagnosis and Therapy; Guangdong Provincial Education Department Key Laboratory of Nano-Immunoregulation Tumor Microenvironment; Central Laboratory, the Second Affiliated Hospital of Guangzhou Medical University, Guangzhou, China; 2 Department of Radiology, the First Affiliated Hospital of Guangzhou Medical University, Guangzhou, China; 3 Department of Pathology, the Second Affiliated Hospital of Guangzhou Medical University, Guangzhou, China; 4 Center for Clinical Trial, the Second Affiliated Hospital of Guangzhou Medical University, Guangzhou, China; 5 Department of Radiology, Shunde Chinese Medicine Hospital, the Affiliated Hospital of Traditional Chinese Medicine University of Guangzhou, Foshan, China

**Keywords:** advanced solid tumors, CT guidance, immunotherapy, intratumoral injection, safety

## Abstract

**Background:**

Systemic administration of immunotherapy via intravenous injection is frequently associated with off-target toxicity throughout the body. In contrast, intratumoral injection has emerged as a promising strategy to mitigate systemic adverse effects. However, data regarding the safety of CT-guided intratumoral immunotherapy remain limited.

**Methods:**

This pooled prospective cohort study included patients from several single-arm clinical trials. Eligible participants had histologically confirmed advanced solid tumors that were refractory or intolerant to standard therapies. Each participant had at least one measurable tumor lesion accessible for puncture under imaging guidance. All patients received CT-guided intratumoral injection of various ICIs (PD-1, PD-L1, and CTLA-4 inhibitors) either alone or in combination, or of CAR-T cells. The primary endpoint was safety of the treatment.

**Results:**

A total of 169 patients were included in the study cohort, with a median follow-up duration of 8.4 months (range, 1.0–38.0 months). Grade 3–4 adverse events occurred in 15 patients (8.88%), comprising 10 (5.92%) grade 3 and 5 (2.96%) grade 4 events; no treatment-related deaths were observed. Efficacy outcomes included 4 patients (2.37%) with complete response (CR), 15 (8.88%) with partial response (PR), 142 (84.02%) with stable disease (SD), and 8 (4.73%) with progressive disease (PD). The objective response rate (ORR) was 11.24%, and the disease control rate (DCR) was 95.27%. The median progression-free survival (PFS) was 3.6 months (95% CI, 3.1–4.1 months), and the median overall survival (OS) was 8.8 months (95% CI, 8.2–9.3 months).

**Conclusion:**

This study indicates the safety and preliminary therapeutic potential of intratumoral injection. Intratumoral injection may be a promising strategy for mitigating systemic toxicity; however, further research is necessary to validate its therapeutic efficacy.

**Clinical trial registration:**

## Introduction

Over the past decade, systemic immunotherapy has demonstrated significant clinical benefits as an adjuvant treatment for solid tumors (1–3). However, routine intravenous delivery may lead to systemic off-target toxicities affecting multiple organs, including pneumonitis, arthritis, and other immune-related adverse events (4, 5). Consequently, increasing attention has been directed toward strategies that deliver immunoregulatory agents directly into tumor lesions using image-guided puncture techniques.

Intratumoral immunotherapy enables a higher local concentration and improved bioavailability of immunotherapeutic agents within the tumor while minimizing systemic exposure and reducing the risk of treatment-related toxicity (6, 7). Moreover, achieving a higher intratumoral drug concentration may reduce the total dose required to trigger effective immune activation, potentially lowering the treatment costs for patients with cancer (8–10). In addition, immunotherapeutic agents can modulate the immunosuppressive tumor microenvironment (TME), converting it into an immunostimulatory microenvironment that promotes tumor-specific immune responses and enhances tumor cell killing (11–13). Activated effector immune cells generated during this process may circulate through the bloodstream and exert antitumor activity at distant, uninjected tumor sites, a phenomenon known as the abscopal effect, or systemic effects following local therapy (14–16).

In 2015, the U.S. Food and Drug Administration (FDA) approved talimogene laherparepvec (T-VEC), the first oncolytic virus-based immunotherapy, for the treatment of patients with unresectable metastatic melanoma (17). Since then, a variety of intratumoral immunotherapeutic agents have been investigated, including RNAs, cytokines, oncolytic viruses, bacteria, monoclonal antibodies, pattern recognition receptor agonists, and immune cells (18–23). Furthermore, early clinical studies of combination intratumoral immunotherapy strategies have demonstrated promising results, suggesting improved local and systemic antitumor efficacy (24–26). Collectively, these studies suggest that local delivery of immunotherapeutic agents into tumors may represent an effective strategy to overcome several limitations associated with systemic drug administration (27–29).

Despite the growing interest in intratumoral immunotherapy, the safety, feasibility, and technical standardization of CT-guided intratumoral injection remain insufficiently studied, and there is currently no consensus regarding standardized procedural protocols. Therefore, in this study, we aimed to evaluate the safety and preliminary efficacy of CT-guided intratumoral delivery of immunotherapeutic agents in patients with advanced solid tumors and to provide clinical evidence that may help inform technical guidelines for intratumoral immunotherapy.

## Methods

### Ethics approval

This study was designed as a pooled analysis of multiple prospective, single-arm, non-randomized, open-label clinical trials conducted at the Second Affiliated Hospital (Panyu Campus) of Guangzhou Medical University in Guangzhou, China. The Ethics Committee of the Second Affiliated Hospital of Guangzhou Medical University approved the study protocol. The clinical trials were registered with ClinicalTrials.gov, and the ClinicalTrials.gov identifiers (NCT number) are provided in **Supplementary Table 1**. The pooled analysis encompasses data collected from March 1, 2018, to December 31, 2024. All participants in the constituent trials received comprehensive study information and provided written informed consent prior to enrollment.

### Study population

Eligible participants were ≥18 years old and had histologically confirmed advanced solid tumors that were refractory to or intolerant of standard therapies. Each participant had at least one measurable tumor lesion that was accessible for puncture under imaging guidance. The estimated life expectancy was at least three months.

The key exclusion criteria were severe organ failure, serious underlying diseases or critical medical conditions, prior treatment similar to the proposed intervention, inability to comply with treatment procedures or follow-up assessments, and a history of severe allergic reactions to any of the drugs used in this study.

### Endpoints

The primary endpoint of this study was safety. Secondary endpoints were progression-free survival (PFS) and overall survival (OS).

### CT-guided intratumoral injection

All procedures were performed by experienced interventional physicians. The puncture needle was selected based on the tumor size and the distance between the tumor center and the puncture entry site on the skin. Under CT guidance, the needle was inserted into the center of the lesion, and the immunotherapeutic agent was administered in accordance with the predetermined dose and injection volume. Successful intratumoral delivery was characterized by the administration of the planned total drug dose within the target tumor. For large lesions, multiple needles were employed to ensure adequate drug distribution.

Decisions regarding treatment for each patient were determined by a multidisciplinary team utilizing pretreatment immunohistochemistry, genetic testing, and the patient’s previous use of immune agents as the basis for their evaluations. For example, patients who had previously received PD-1 antibodies with no clinical response may be treated with PD-L1 antibodies.

All patients received CT-guided intratumoral injections of specific immunotherapeutic agents, which included PD-1 inhibitors (such as pembrolizumab, nivolumab, and sintilimab), PD-L1 inhibitors (such as atezolizumab and durvalumab), and CTLA-4 inhibitors (such as ipilimumab), either as monotherapy or in combination. The intratumoral dosage for each immune checkpoint inhibitor (ICI) was standardized to half of the approved intravenous dosage, as indicated in the drug label. For patients undergoing CAR-T cell therapy, a dose of 1 × 10^6^ cells per kilogram was administered intratumorally. The injection volume was adjusted according to the size of the lesion. Treatments were scheduled every three weeks (Q3W) and continued until disease progression, the occurrence of unacceptable toxicity, or patient withdrawal.

All procedures were performed under local anesthesia with 1% lidocaine to ensure appropriate analgesia. Patients’ vital signs were continuously monitored throughout the procedure. Preoperative planning, intraoperative needle placement, and postoperative evaluation of drug distribution were performed under CT guidance. To visualize intratumoral drug distribution, a 10-fold dilution of an iodine-containing contrast agent, such as ioversol or iodixanol, was combined with selected immunotherapy agents.

### Safety and clinical efficacy evaluation

Safety evaluation involved monitoring adverse events (AEs) and grading them in accordance with the Common Terminology Criteria for Adverse Events (CTCAE), version 5.0. At each follow-up visit, all AEs were documented and assessed for their association with either the intratumoral injection procedure or the immunotherapeutic agent.

The clinical data collected included physical examination findings, ECOG performance status, vital signs, and laboratory tests. Tumor evaluations were performed approximately every three weeks using the same imaging modality and measurement methods to ensure consistency.

Treatment response was assessed according to the Response Evaluation Criteria in Solid Tumors (RECIST) version 1.1. Responses were classified as complete response (CR), partial response (PR), stable disease (SD), or progressive disease (PD). The objective response rate (ORR) was defined as the proportion of patients achieving CR or PR, while the disease control rate (DCR) was defined as the proportion achieving CR, PR, or SD as their best overall response. PFS was defined as the interval from the intratumoral injection to the first documented instance of radiographic disease progression or death from any cause, whichever occurred first. OS was defined as the time from intratumoral injection to death from any cause.

### Statistical analysis

Statistical analyses and survival curves were generated using GraphPad Prism 10 (version 10.1.2). The median follow−up duration was estimated using the reverse Kaplan–Meier method. Continuous variables that did not follow a normal distribution were presented as medians (ranges) with 95% confidence intervals (CIs), whereas categorical variables were expressed as percentages. Given the single−arm, exploratory design and the heterogeneity of immunotherapeutic agents and tumor types across this pooled cohort, all analyses were descriptive, and no formal comparisons were made between trials or treatment subgroups.

## Results

### Participants and baseline characteristics

A total of 202 patients were initially screened for eligibility. Of these, 33 patients underwent only puncture biopsy and were excluded from the final analysis. Ultimately, 169 patients were included in the pooled cohort. All data reported in this study were collected and analyzed prior to March 31, 2025. The demographic and baseline disease characteristics of the patients are summarized in **Table 1**. At the data cutoff, 28 patients (16.6%) remained under active follow-up, whereas 141 patients (83.4%) had reached their endpoints. The median follow-up duration was 8.4 months, ranging from 1.0 to 38.0 months.

**Table 1:** Demographic and disease characteristics at baseline.

Characteristics	Patients (N = 169)
Age, years	
Mean	55
Range	20-87
Age category, n (%)	
<65 years	116 (68.64)
≥65 years	53 (31.36)
Sex, n (%)	
Male	113 (66.86)
Female	56 (33.14)
ECOG performance status score, n (%)	
0	68 (40.24)
1	51 (30.18)
2	34 (20.12)
3	16 (9.46)
Cancer histological distribution, n (%)	
Lung cancer	45 (26.63)
Liver cancer	41 (24.26)
Gastrointestinal cancer	30 (17.75)
Pancreatic cancer	16 (9.47)
Urinary tract cancer	5 (2.96)
Breast cancer	5 (2.96)
Oral squamous cell cancer	4 (2.37)
Ovarian cancer	4 (2.37)
Malignant melanoma of the skin	3 (1.78)
Vaginal cancer	2 (1.18)
Others	14 (8.27)
Metastasis, n (%)	
Yes	130 (76.92)
No	39 (23.08)
Number of intratumoral injections, n (%)	
1	55 (32.54)
2	48 (28.40)
3	27 (15.98)
4	18 (10.65)
≥5	21 (12.43)

ECOG, Eastern Cooperative Oncology Group.

ECOG performance status scores range from 0 to 5, with 0 indicating no.

symptoms and higher scores indicating increasing disability.

The histological distribution of advanced solid tumors comprised 45 patients (26.63%) with lung cancer, 41 patients (24.26%) with liver cancer, and 30 patients (17.75%) with gastrointestinal cancer. Additionally, there were 16 patients (9.47%) with pancreatic cancer, 5 patients (2.96%) with urinary tract cancer, and 5 patients (2.96%) with breast cancer. Furthermore, 4 patients (2.37%) had oral squamous cell carcinoma, 4 patients (2.37%) had ovarian cancer, and 3 patients (1.78%) had cutaneous malignant melanoma. The cohort also included 2 patients (1.18%) with vaginal cancer and 14 patients (8.27%) with other types of tumors.

To comprehensively evaluate the safety and efficacy of intratumoral injection therapy, exploratory subgroup analyses were stratified by tumor type and treatment regimen. A minimum reporting threshold of 10 cases per category was established for both stratification approaches. Four tumor-type subgroups met this threshold: lung cancer (n=45), liver cancer (n=41), gastrointestinal cancer (n=30), and pancreatic cancer (n=16). Similarly, three treatment regimens met this threshold: PD-1/CTLA-4 inhibitors (n=75), PD-L1/CTLA-4 inhibitors (n=53), and CAR-T cells (n=21). The remaining tumor types and treatment groups were excluded because of insufficient sample sizes. No formal statistical comparisons were conducted between the groups in either analysis, given the descriptive nature of these exploratory analyses and the disparity in sample sizes.

### Intratumoral injection procedures

During the study period, 169 patients underwent 878 CT-guided intratumoral injection procedures. The key characteristics of these procedures are summarized in **Table 2**. Some patients received multiple injections, and in certain treatment sessions, up to five different tumor sites were punctured within a single procedure. The most frequently used needle gauges were 21G and 23G.

**Table 2:** Characteristics of intratumoral injection procedures.

Characteristics	Procedures (N = 878)
Target lesion length (cm)	
Mean	3.7
Range	1.0-31.5
Needle size, n (%)	
17G, 18G, 19G and 20G	78 (8.88)
21G	359 (40.89)
22G	38 (4.33)
23G	403 (45.90)
Sites of injection, n (%)	
Liver	264 (30.07)
Abdominal cavity	115 (13.10)
Lung	112 (12.76)
Subcutaneous	86 (9.80)
Retroperitoneal	84 (9.57)
Osteoarticular and vertebral	68 (7.74)
Head and neck	54 (6.15)
Pelvic cavity	42 (4.78)
Mediastinum and pleura	35 (3.99)
Pancreas	18 (2.04)
Lesion type, n (%)	
Metastatic	615 (70.05)
Primary	263 (29.95)
Drug leakage, n (%)	
Yes	106 (12.07)
No	772 (87.93)

Among the 878 intratumoral injection procedures, the distribution of target lesions was as follows: liver lesions (264, 30.07%), abdominal cavity lesions (115, 13.10%), lung lesions (112, 12.76%), subcutaneous lesions (86, 9.80%), retroperitoneal lesions (84, 9.57%), osteoarticular and vertebral lesions (68, 7.74%), head and neck lesions (54, 6.15%), pelvic cavity lesions (42, 4.78%), mediastinal and pleural lesions (35, 3.99%), and pancreatic lesions (18, 2.04%).

Drug leakage into the surrounding interstitial tissue was observed in 106 of the 878 intratumoral injection procedures (12.07%). In the majority of these cases, leakage was confined to the peritumoral area and did not necessitate any additional intervention. Furthermore, no severe complications, such as organ infarction or major bleeding, were directly attributed to the leakage.

### Safety

Adverse events (AEs) were systematically monitored and documented throughout the follow-up period following each intratumoral injection, as detailed in **Table 3**. Among the 169 patients, the most frequently reported events were fever (102, 60.36%), local pain at the puncture site (92, 54.44%), chills (31, 18.34%), and vomiting (25, 14.79%). The majority of these adverse events were mild and resolved spontaneously within 24 hours, without necessitating specific medical intervention. For patients who experienced intolerable symptoms, symptomatic treatment was sufficient to relieve the adverse effects.

**Table 3:** Treatment-related adverse events observed during follow-up.

Adverse events	Patients (N = 169)	
	Any grade, n (%)	Grade 3–4, n (%)
Fever	102 (60.36)	13 (7.69)
Local pain at the puncture site	92 (54.44)	7 (4.14)
Chills	31 (18.34)	4 (2.37)
Vomiting	25 (14.79)	3 (1.78)
Leukocytopenia	16 (9.47)	1 (0.59)
Fatigue	13 (7.69)	2 (1.18)
Nausea	13 (7.69)	2 (1.18)
Alanine aminotransferase increased	13 (7.69)	0
Cough	8 (4.73)	1 (0.59)
Dyspnea	7 (4.14)	2 (1.18)
Hypotension	5 (2.96)	5 (2.96)
Pruritus	4 (2.37)	0
Immune-related pneumonitis	4 (2.37)	4 (2.37)
Dizziness	3 (1.78)	0
Immune-related rash	3 (1.78)	3 (1.78)
Respiratory failure	3 (1.78)	3 (1.78)
Diarrhea	2 (1.18)	0

All participants who received at least one trial treatment were included in the analyses.

Adverse events attributed to the treatment were documented by the investigators on the case report form.

According to the Common Terminology Criteria for Adverse Events (CTCAE) version 5.0, 15 patients (8.88%) experienced grade 3–4 AEs. Among these, 10 patients (5.92%) had grade 3 AEs, while 5 patients (2.96%) experienced grade 4 AEs. Grade 3 AEs included hypotension requiring hospitalization (3/169, 1.78%), immune-related rash (3/169, 1.78%), and immune-related pneumonitis necessitating oxygen and corticosteroids (4/169, 2.37%). Grade 4 AEs included shock due to severe hypotension (2/169, 1.18%) and respiratory failure (3/169, 1.78%). Importantly, no treatment-related deaths (grade 5 AEs) were noted. No significant association was observed between the incidence or type of AEs and tumor histological type.

In subgroup analyses, grade 3–4 adverse events were observed in 5 of 45 patients (11.11%; 95% CI, 3.71%–24.05%) with lung cancer, 3 of 41 (7.32%; 95% CI, 1.54%–19.92%) with liver cancer, 2 of 30 (6.67%; 95% CI, 0.82%–22.07%) with gastrointestinal cancer, and 1 of 16 (6.25%; 95% CI, 0.16%–30.23%) with pancreatic cancer. By treatment regimen, grade 3–4 adverse events were reported in 7 of 75 patients (9.33%; 95% CI, 3.84%–18.29%) receiving PD-1/CTLA-4 inhibitors, 6 of 53 (11.32%; 95% CI, 4.27%–23.03%) receiving PD-L1/CTLA-4 inhibitors, and 1 of 21 (4.76%; 95% CI, 0.12%–23.82%) receiving CAR-T cells. Overall, the incidence of severe toxicities was low across all subgroups, with wide confidence intervals reflecting limited sample sizes.

### Clinical response

**Figure 1:**
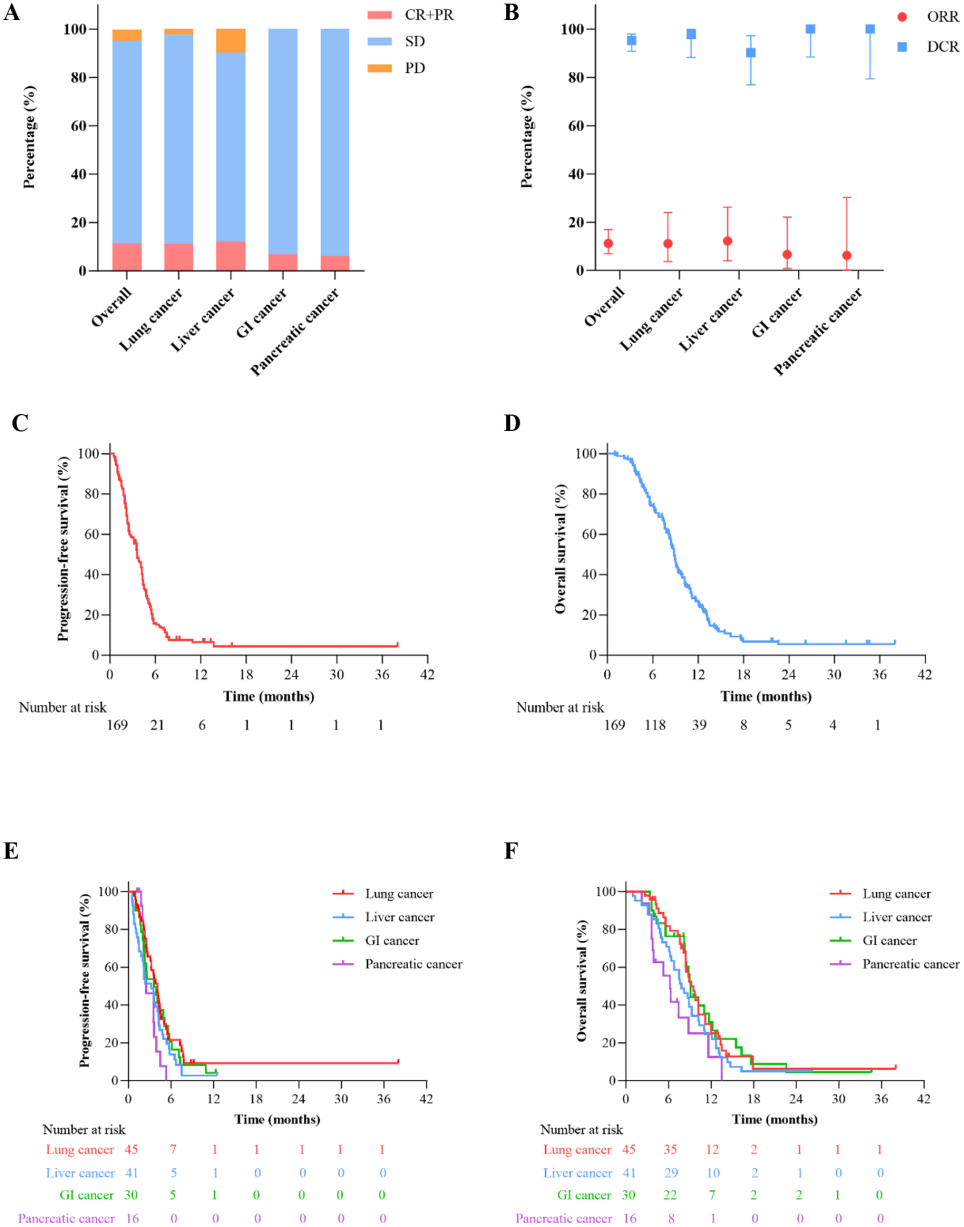
Treatment response and survival outcomes. **(A)** Best overall response distribution (CR+PR, SD, and PD) in the overall cohort and by cancer type. **(B)** ORR and DCR with 95% confidence intervals (error bars) in the overall cohort and according to cancer type. **(C, D)** Kaplan-Meier estimates of PFS and OS in the overall cohort. **(E, F)** PFS and OS stratified by cancer type. The numbers at risk are shown below the survival curves. CR, complete response; PR, partial response; SD, stable disease; PD, progressive disease; ORR, objective response rate; DCR, disease control rate; PFS, progression-free survival; OS, overall survival; GI, gastrointestinal.

After the initial treatment, therapeutic efficacy was evaluated using contrast-enhanced CT imaging every three weeks until patient withdrawal or disease progression. The results showed that 4 patients (2.37%) achieved complete response (CR), 15 (8.88%) achieved partial response (PR), 142 (84.02%) had stable disease (SD), and 8 (4.73%) experienced progressive disease (PD) (**Figure 1A**). The objective response rate (ORR) was 11.24% (19/169), and the disease control rate (DCR) was 95.27% (161/169) based on the best overall response (**Figure 1B**). To further evaluate these efficacy outcomes, a descriptive analysis was conducted on patients who received at least two sessions of intratumoral injections (n=114). In this analysis, the ORR was found to be 15.79% (18/114) and the DCR was 93.86% (107/114), which were generally consistent with the overall cohort findings.

The median PFS was 3.6 months (95% CI, 3.1–4.1 months), with 6-month and 12-month PFS rates of 15.9% and 6.5%, respectively. The median OS was 8.8 months (95% CI, 8.2–9.3 months), with 6-month and 12-month OS rates of 74.2% and 25.5%, respectively. The Kaplan-Meier curves for PFS and OS are illustrated in **Figures 1C, D**. As shown in **Figure 1C**, the PFS curve demonstrated a steep decline during the first 6 months, with only a modest number of additional events observed thereafter. In contrast, the OS curve (**Figure 1D**) showed a more gradual decline, with the majority of survival events occurring between 6 and 18 months.

To provide a preliminary overview of efficacy across tumor types, the clinical outcomes for the four subgroups were descriptively summarized (**Figures 1A, B**; **Supplementary Table 2**). The ORRs for lung, liver, gastrointestinal, and pancreatic cancers were 11.11% (95% CI, 3.71%–24.05%), 12.20% (95% CI, 4.08%–26.20%), 6.67% (95% CI, 0.82%–22.07%), and 6.25% (95% CI, 0.16%–30.23%), respectively. The median PFS and OS for each subgroup are illustrated in the corresponding Kaplan-Meier curves (**Figures 1E, F**). Notably, the DCR remained elevated across all subgroups, ranging from 90.24% to 97.78%, despite variations in objective response rates.

Similarly, clinical outcomes for the three treatment regimen groups were descriptively summarized (**Supplementary Table 3**). Modest ORRs (9.43%–14.67%) and high DCRs (90.48%–97.33%) were observed, with the median PFS and OS ranging from 2.6 to 4.2 months and 7.6 to 8.9 months. In analyses of both tumor types and treatment regimens, the broad confidence intervals observed in smaller subgroups highlight the statistical uncertainty inherent in the limited sample sizes.

Interestingly, 36 patients (21.30%) demonstrated shrinkage or disappearance of non-target lesions. Among these patients, 8 (4.73%) received injections targeting primary tumors, 16 (9.47%) received injections targeting metastatic lesions, and 12 (7.10%) received injections targeting both primary and metastatic lesions.

### Case reports

**Figure 2:**
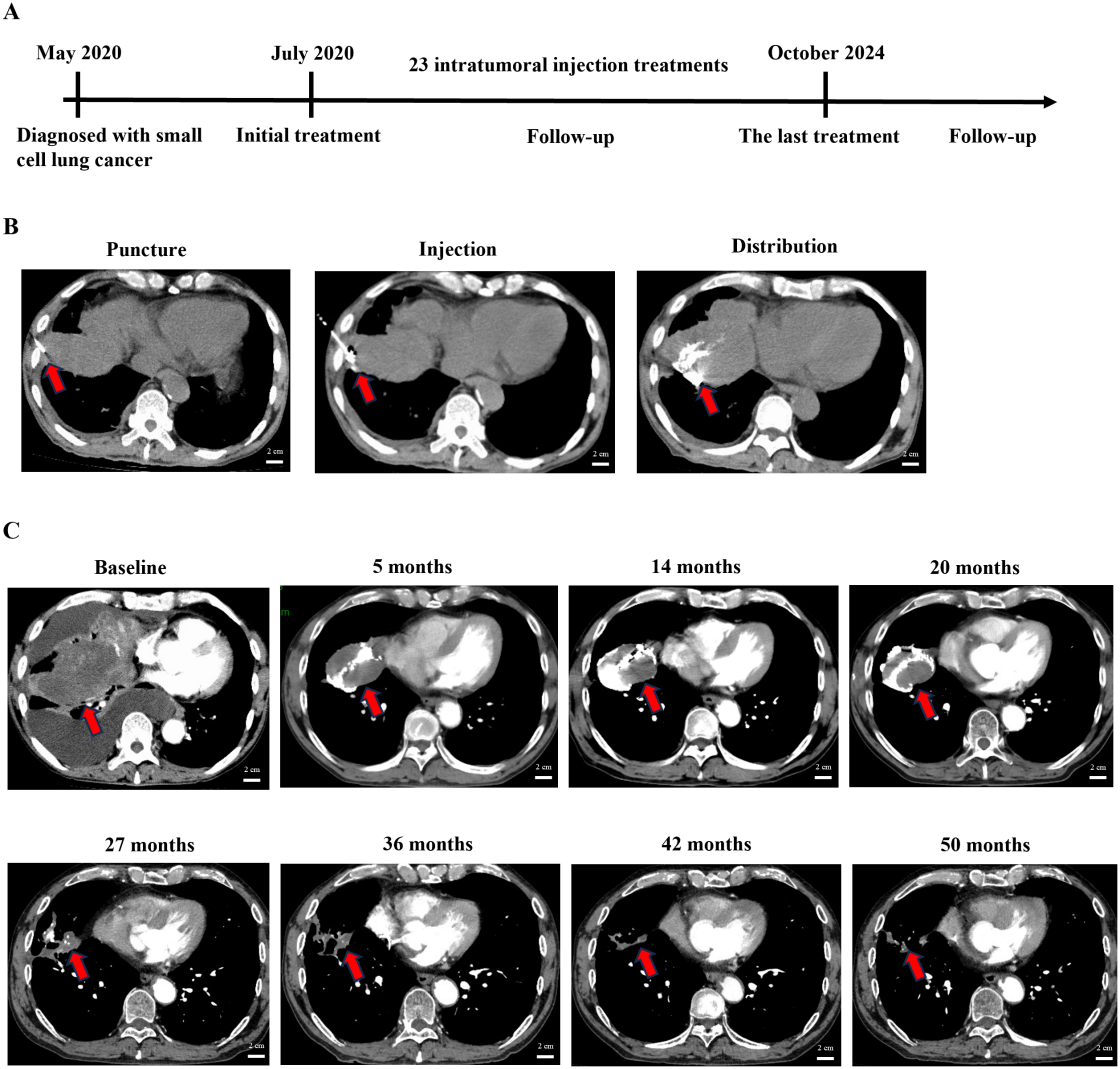
Treatment timeline and radiological follow-up of an elderly male patient with small cell lung cancer (SCLC). **(A)** Schematic overview of the treatment course. **(B)** CT images depicting the puncture and injection procedure for the right lung tumor. **(C)** Contrast-enhanced CT images revealing a series of changes in the right lung lesion (red arrows) throughout the treatment course.

An elderly man was diagnosed with small cell lung cancer (SCLC) via biopsy in May 2020. Despite initial systemic immunotherapy, the lesion progressed rapidly. The initial intratumoral injection was administered in July 2020, and the final treatment was administered in October 2024 (**Figure 2A**). Throughout this period, a total of 23 treatments were performed, targeting the tumor in the right lung for each intratumoral injection (**Figure 2B**). Compared with the baseline, the tumor in the right lung began to shrink by the 5th month and demonstrated significant regression by the 27th month (**Figure 2C**). Subsequently, the lesion continued to regress, and the degree of regression correlated with the cumulative number of treatments.

### Regression of non-target lesions

**Figure 3:**
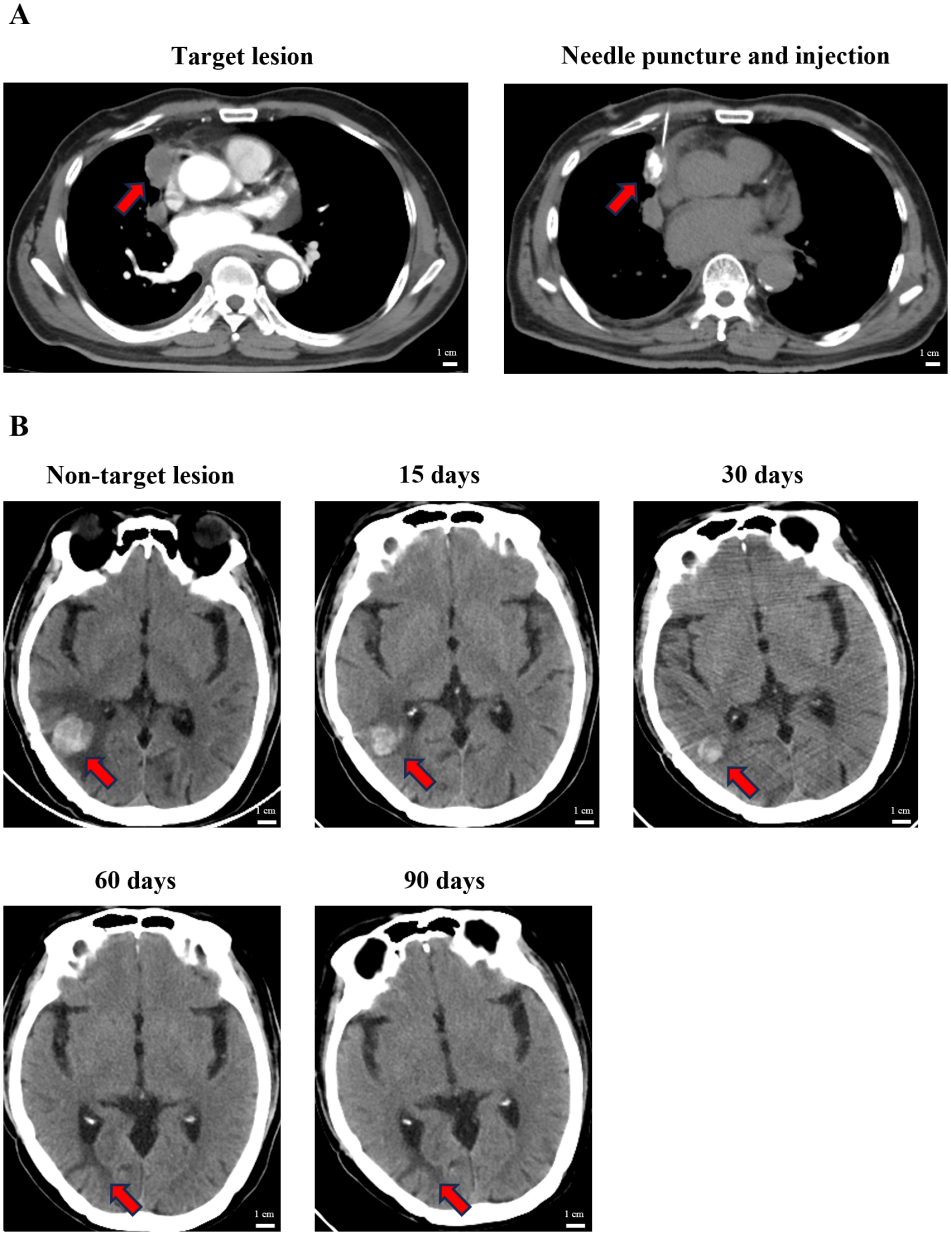
CT-guided intratumoral injection of the primary lung lesion and serial radiological follow-up of the untreated brain metastasis. **(A)** CT images of the target lung lesion before treatment (red arrow) and during percutaneous needle puncture and intratumoral injection (red arrow). **(B)** Serial CT images of an untreated non-target brain metastasis in the right occipital lobe, demonstrating progressive regression at baseline (pre-treatment) and at 15, 30, 60, and 90 days post-treatment (red arrows).

In a notable case, a patient diagnosed with right lung cancer and concurrent intracranial metastasis underwent CT-guided intratumoral injection targeting the lung lesion (**Figure 3A**). Serial follow-up CT imaging demonstrated progressive regression of the untreated brain metastasis (**Figure 3B**). Remarkably, this distant lesion had not received direct injection or any other form of localized therapy. These findings suggest a systemic antitumor response potentially associated with localized intratumoral immunotherapy.

## Discussion

Intratumoral injection has long been proposed as a potential strategy to enhance the therapeutic efficacy of immunotherapy. However, to date, the applicability, safety, and clinical outcomes of intratumoral injection techniques remain insufficiently characterized. In particular, there is a lack of clinical data evaluating the safety and efficacy of intratumoral delivery of immunotherapeutic agents in patients with advanced solid tumors.

According to the CTCAE version 5.0, the majority of the AEs observed in this study were classified as grade 1-2. Grade 3–4 AEs occurred in only 15 patients (8.88%), with no treatment-related deaths (grade 5 AEs) reported. Tebotelimab, a PD-1 and LAG-3 antagonist, showed an ORR of 7% (95% CI, 3.8–12.2) in solid tumors, with 22% of patients experiencing grade ≥3 treatment-related adverse events (TRAEs) (30). Among patients with advanced or metastatic solid tumors receiving nivolumab plus ipilimumab, grade 3–4 AEs occurred in 28.7% of patients with high tumor tissue mutational burden (tTMB-H) and 37.3% of patients with high circulating tumor DNA mutational burden (bTMB-H) (31). Compared with these studies, our results suggest that CT-guided intratumoral immunotherapy may be associated with a lower incidence of severe adverse events, although the improvement in response rate remains modest.

The absence of a contemporaneous comparator arm receiving systemic administration in our study precludes a definitive causal inference regarding the incremental safety benefits of the intratumoral approach. Furthermore, in the subgroup analysis, the incidence of grade 3–4 adverse events remained low across all subgroups (6.25%–11.32%), whereas the disease control rate was notably high (90.24%–97.78%). These findings suggest that CT-guided intratumoral immunotherapy achieves a high disease control rate with a favorable safety profile. However, it is important to note that this disease control was primarily driven by stable disease, and the modest objective response rate (11.24%) along with a short median progression-free survival (3.6 months) indicates that the therapeutic efficacy remains limited. Therefore, these results should be interpreted as proof-of-concept rather than definitive evidence of antitumor efficacy.

In addition to local tumor control, intratumoral immunotherapy may elicit systemic antitumor immune responses. However, because peripheral blood immune correlates were not assessed in this study, the observed shrinkage of non-target lesions could stem from multiple factors and cannot be mechanistically attributed to a systemic immune effect of the local therapy based solely on the current data. Furthermore, the modest sample sizes across both tumor−type (16–45 patients) and treatment−regimen (21–75 patients) subgroups limited the precision of the efficacy estimates and precluded formal statistical comparisons.

Several limitations must be acknowledged. The heterogeneity of immunotherapeutic agents within the cohort precludes the definitive attribution of outcomes solely to the intratumoral delivery modality. This pooled cohort study was designed to evaluate the feasibility and preliminary efficacy of intratumoral injection as a universal platform, rather than to compare different agents; thus, the non-randomized, exploratory design inherently prevents head-to-head comparisons. Consequently, the observed outcomes likely reflect intrinsic differences in drug potency, baseline characteristics, tumor biology, and delivery strategy. Furthermore, the individualized selection of agents and limited sample size further restrict the generalizability of the efficacy findings. Additionally, as a pooled analysis of multiple independent trials with heterogeneous regimens, our findings should be considered hypothesis-generating and warrant validation in larger homogeneous prospective studies.

In general, our study suggests that CT-guided intratumoral immunotherapy is a feasible and relatively safe treatment strategy for patients with advanced solid tumors. Nevertheless, further studies with larger cohorts and optimized treatment strategies are required to determine the clinical benefits of this approach for different tumor types.

## Data Availability

The original contributions presented in the study are included in the article/**Supplementary Material**. Further inquiries can be directed to the corresponding author/s.
