# Supplementary material for: CT-guided intratumoral immunotherapy for advanced solid tumors: a prospective clinical study of safety and systemic antitumor effects
Source: Front Immunol. 2026 Jul 24;17:1869154. doi: 10.3389/fimmu.2026.1869154 (PMC13447376; doi:10.3389/fimmu.2026.1869154)
Supplement: Supplementary file 1 [file Table1.docx]

**Supplementary Table S1. Summary of our clinical trials for intratumoral injection projects registered at ClinicalTrials.gov.**

| ClinicalTrials.gov ID | Trial Title | Intratumoral Injection Product | Phase | Start Date | Enrolled patients (n) |
| --- | --- | --- | --- | --- | --- |
| NCT03198052 | GPC3/Mesothelin/Claudin18.2/GUCY2C/B7H3/PSCA/PSMA/MUC1/TGFβ/HER2/Lewis-Y/AXL/EGFR-CAR-T Cells against cancers | CAR-T cells | I | 7/1/2017 | 18 |
| NCT03769129 | Evaluating the safety and efficacy of Pembrolizumab combined with MWA for advanced NSCLC | Pembrolizumab | II | 11/1/2018 | 6 |
| NCT03755739 | Trans-artery/intra-tumor infusion of checkpoint inhibitors plus chemodrug for immunotherapy of advanced solid tumors | Checkpoint inhibitors (CPIs) | II | 11/1/2018 | 134 |
| NCT03952065 | IT or IA infusion of PD1/PDL1/CTLA4 inhibitors versus IV for immunotherapy of head/neck cancers (HNC) | PD1/PDL1/CTLA4 inhibitors | II | 1/1/2019 | 8 |
| NCT05341492 | EGFR/B7H3 CAR-T on lung cancer and triple negative breast cancer | CAR-T cells | I | 5/1/2022 | 3 |

Abbreviations: MWA, microwave ablation; IT, intra-tumor; IA, intra-artery; IV, intravenous.

**Supplementary Table S2. Efficacy outcomes by tumor type in the subgroup analysis.**

| Tumor Type | n | ORR, n(%) | DCR, n(%) | mPFS, months (95% CI) | mOS,  months  (95% CI) |
| --- | --- | --- | --- | --- | --- |
| Lung cancer | 45 | 5 (11.11) | 44 (97.78) | 4.1 (2.7-4.5) | 9.5 (8.4-10.2) |
| Liver cancer | 41 | 5 (12.20) | 37 (90.24) | 3.2 (1.8-4.2) | 7.8 (6.2-9.3) |
| Gastrointestinal cancers | 30 | 2 (6.67) | 29 (96.67) | 3.6 (2.2-5.1) | 9.1 (8.3-11.7) |
| Pancreatic cancer | 16 | 1 (6.25) | 15 (93.75) | 2.5 (2.1-3.6) | 6.2 (3.6-8.8) |
| Other solid tumors* | 37 | - | - | - | - |

* Includes urinary tract cancers (n=5), breast cancer (n=5), oral squamous cell carcinoma (n=4), ovarian cancer (n=4), cutaneous malignant melanoma (n=3), vaginal cancer (n=2), and other miscellaneous tumors (n=14).

**Supplementary Table S3. Efficacy outcomes by treatment regimen in the subgroup analysis.**

| Treatment Regimen | n | ORR, n(%) | DCR, n(%) | mPFS, months (95% CI) | mOS,  months  (95% CI) |
| --- | --- | --- | --- | --- | --- |
| PD-1/CTLA-4 inhibitors | 75 | 11 (14.67) | 73 (97.33) | 3.5 (2.5–4.3) | 8.9 (8.1–9.9) |
| PD-L1/CTLA-4 inhibitors | 53 | 5 (9.43) | 51 (96.23) | 2.6 (2.3–4.5) | 8.8 (8.2–10.8) |
| CAR-T cells | 21 | 2 (9.52) | 19 (90.48) | 4.2 (3.2–4.9) | 7.6 (5.1–9.7) |

Other regimens (n=20) were not analyzed separately due to heterogeneous treatment protocols and insufficient sample size for individual regimen categories.
